# Supplementary material for: Transcriptional Changes in Radiation-Induced Lung Injury: A Comparative Analysis of Two Radiation Doses for Preclinical Research
Source: Int J Mol Sci. 2024 Mar 28;25(7):3766. doi: 10.3390/ijms25073766 (PMC11011446; doi:10.3390/ijms25073766)
Supplement: Supplementary file 1 [file ijms-25-03766-s001.zip › ijms-2875567-supplementary.pdf]

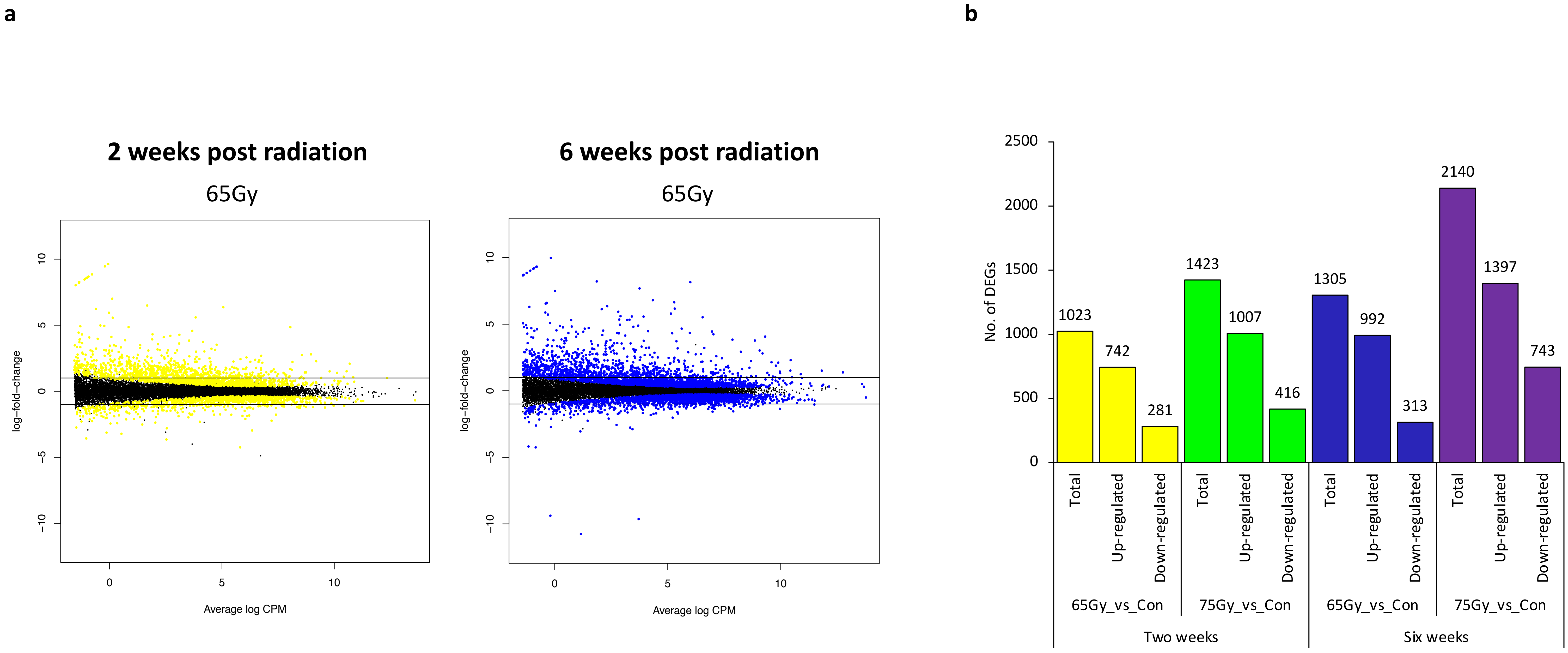

Figure S1 Differentially expressed genes (DEGs) of 65 Gy- and 75 Gy-irradiated lung tissues. (**a**) Mean difference (MD) plots showing log-fold change and average abundance of each gene at the inflammation and fibrosis stages. The black line is a threshold to distinguish genes with a log-fold change greater than 1. (**b**) barplot showing the number of DEGs.

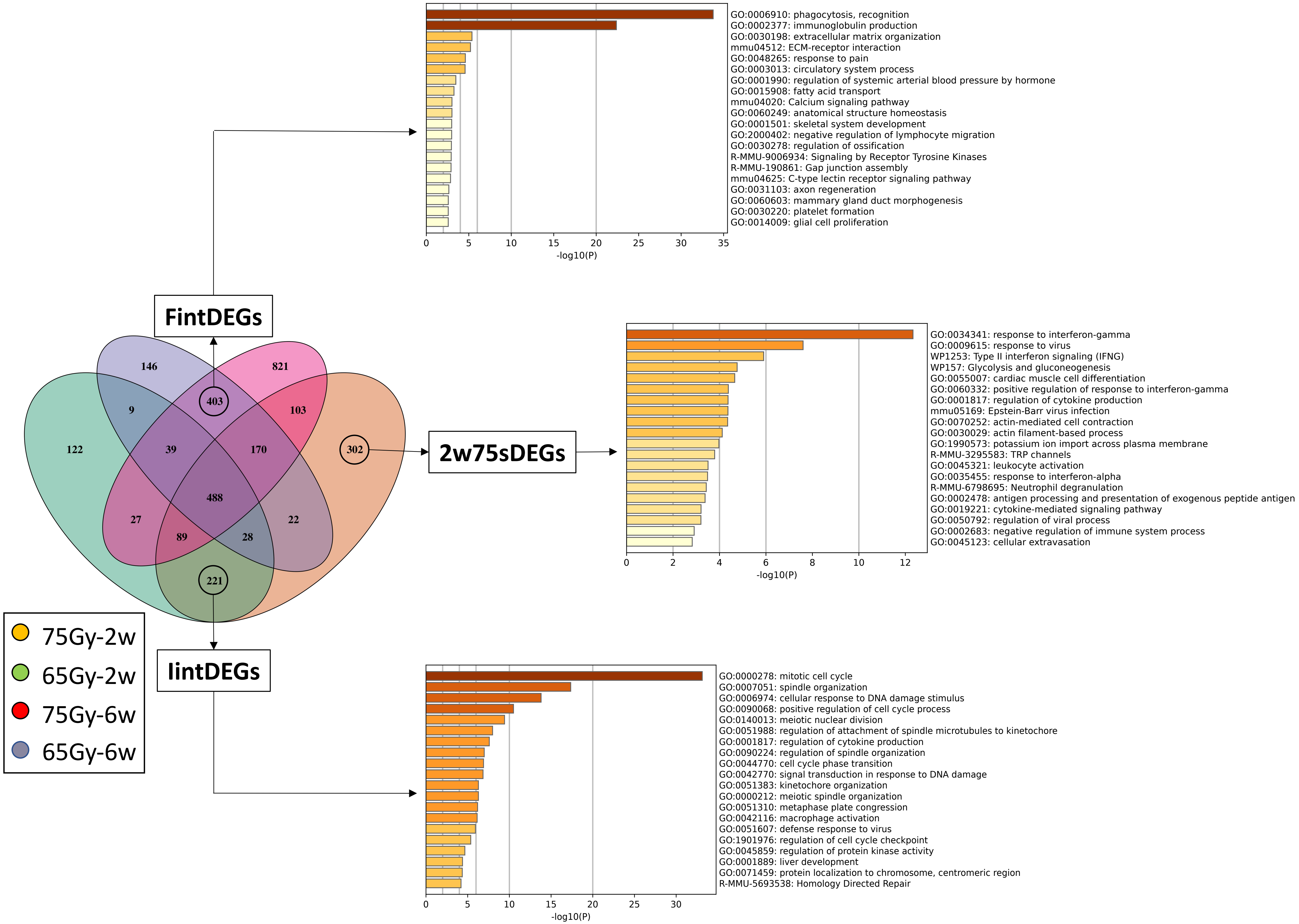

Figure S2 Investigation of dose-intersected and specific differentially expressed genes (DEGs). Venn Diagram displaying the 65 Gy- and 75 Gy-intersected DEGs at the inflammation and fibrosis stages, namely IintDEGs and FintDEGs, respectively, as well as 75Gy-specific DEGs at the inflammation stage, namely 2w75sDEGs, with corresponding gene ontology (GO) enrichment and Kyoto Encyclopedia of Genes and Genomes (KEGG) pathway. Abbreviations: IintDEGs, inflammation-intersected DEGs; FintDEGs, fibrosis-intersected DEGs; 2w75sDEGs, 2-week-75-specific DEGs.

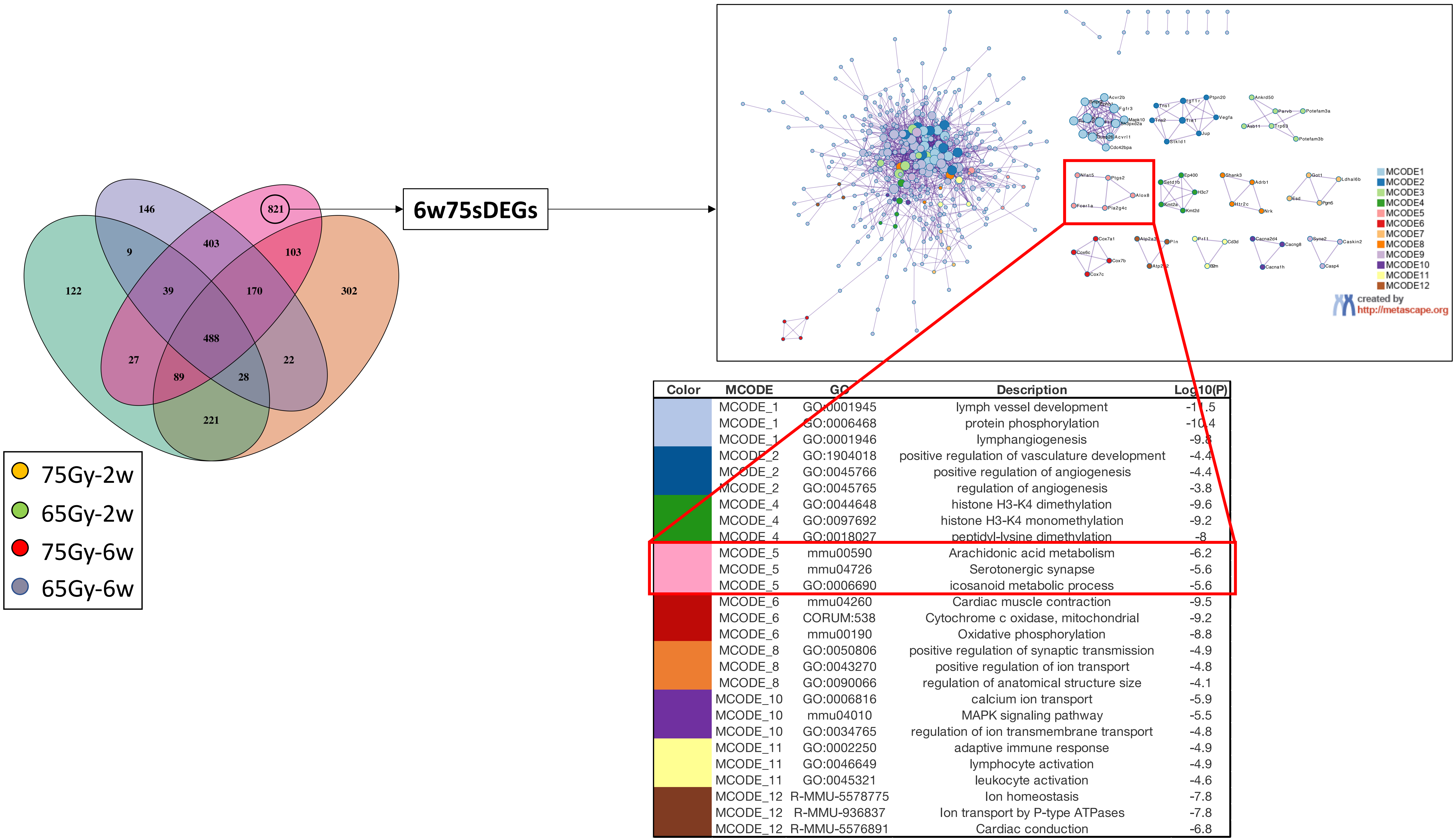

Color	MCODE	GO	Description	Log10(P)
	MCODE_1	GO:0001945	lymph vessel development	-11.5
	MCODE_1	GO:0006468	protein phosphorylation	-10.4
	MCODE_1	GO:0001946	lymphangiogenesis	-9.8
	MCODE_2	GO:1904018	positive regulation of vasculature development	-4.4
	MCODE_2	GO:0045766	positive regulation of angiogenesis	-4.4
	MCODE_2	GO:0045765	regulation of angiogenesis	-3.8
	MCODE_4	GO:0044648	histone H3-K4 dimethylation	-9.6
	MCODE_4	GO:0097692	histone H3-K4 monomethylation	-9.2
	MCODE_4	GO:0018027	peptidyl-lysine dimethylation	-8
	MCODE_5	mmu00590	Arachidonic acid metabolism	-6.2
	MCODE_5	mmu04726	Serotonergic synapse	-5.6
	MCODE_5	GO:0006690	icosanoid metabolic process	-5.6
	MCODE_6	mmu04260	Cardiac muscle contraction	-9.5
	MCODE_6	CORUM:538	Cytochrome c oxidase, mitochondrial	-9.2
	MCODE_6	mmu00190	Oxidative phosphorylation	-8.8
	MCODE_8	GO:0050806	positive regulation of synaptic transmission	-4.9
	MCODE_8	GO:0043270	positive regulation of ion transport	-4.8
	MCODE_8	GO:0090066	regulation of anatomical structure size	-4.1
	MCODE_10	GO:0006816	calcium ion transport	-5.9
	MCODE_10	mmu04010	MAPK signaling pathway	-5.5
	MCODE_10	GO:0034765	regulation of ion transmembrane transport	-4.8
	MCODE_11	GO:0002250	adaptive immune response	-4.9
	MCODE_11	GO:0046649	lymphocyte activation	-4.9
	MCODE_11	GO:0045321	leukocyte activation	-4.6
	MCODE_12	R-MMU-5578775	Ion homeostasis	-7.8
	MCODE_12	R-MMU-936837	Ion transport by P-type ATPases	-7.8
	MCODE_12	R-MMU-5576891	Cardiac conduction	-6.8

Figure S3 Investigation of dose-specific differentially expressed genes (DEGs). Venn Diagram displaying the 75Gy-specific DEGs at the fibrosis stage, namely 6w75sDEGs, with corresponding protein-protein interaction (PPI) network and gene ontology (GO) enrichment and Kyoto Encyclopedia of Genes and Genomes (KEGG) using Metascape. Abbreviations: 2w75sDEGs, 2-week-75-specific DEGs.

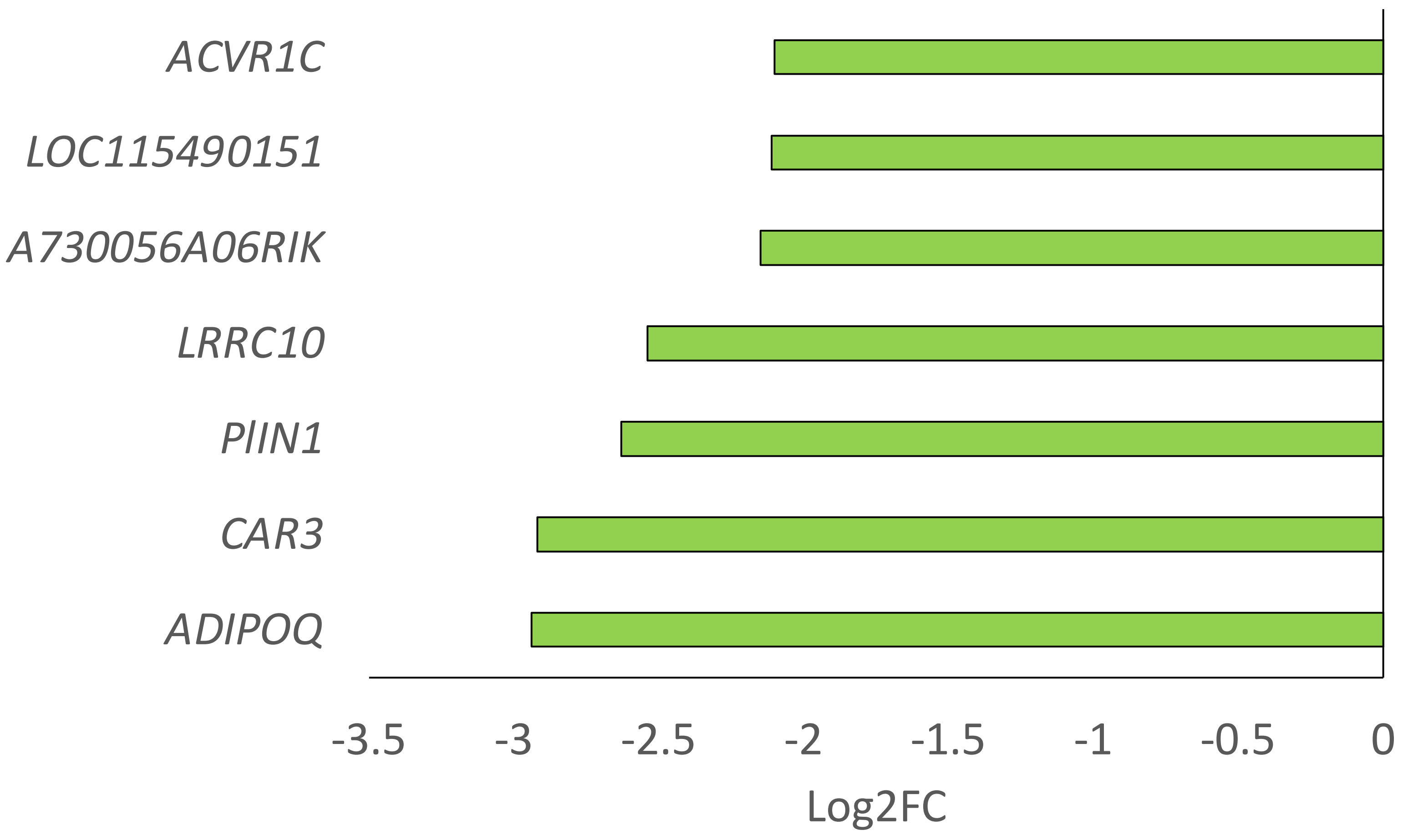

Figure S4 Expression of the top differentially expressed genes (DEGs) of 2w75sDEGs. Abbreviations: 2w75sDEGs, 2-week-75-specific DEGs; Log2FC, Log2 fold change. Threshold for deciding top expressed DEGs is Log2FC=2/-2 & Padj=0.001.

Inflammation score

Score	Description
0	No infiltration of inflammatory cells
1	Occasionally by vein and bronchus cuff inflammatory cells infltration
2	The majority of vein, peribronchial infiltration of inflammatory cells, inflammatory cell layer is 1-5
3	The majority of vein, peribronchial infiltration of inflammatory cells, inflammatory cell layer is greater than 5

Modified Ashcroft score

Score	Description
0	**Alveolar septa:** No fibrotic burden at the most flimsy small fibers in some alveolar walls<br>**Lung structure:** Normal lung
1	**Alveolar septa:** Isolated gentle fibrotic changes (septum ≤3× thicker than normal)<br>**Lung structure:** Alveoli partly enlarged and rarefied, but no fibrotic masses present
2	**Alveolar septa:** Clearly fibrotic changes (septum >3× thicker than normal) with knot-like formation but not connected to each other<br>**Lung structure:** Alveoli partly enlarged and rarefied, but no fibrotic masses
3	**Alveolar septa:** Contiguous fibrotic walls (septum >3× thick- er than normal) predominantly in whole microscopic field **Lung structure:** Alveoli partly enlarged and rarefied, but no fibrotic masses
4	**Alveolar septa:** Variable<br>**Lung structure:** Single fibrotic masses (≤10% of micro- scopic field)
5	**Alveolar septa:** Variable<br>**Lung structure:** Confluent fibrotic masses (>10% and ≤50% of microscopic field). Lung structure severely dam- aged but still preserved
6	**Alveolar septa:** Variable, mostly not existent<br>**Lung structure:** Large contiguous fibrotic masses (>50% of microscopic field). Lung architecture mostly not preserved
7	**Alveolar septa:** Non-existent<br>**Lung structure:** Alveoli nearly obliterated with fibrous masses but still up to five air bubbles
8	**Alveolar septa:** Non-existent<br>**Lung structure:** Microscopic field with complete obliteration with fibrotic masses

Figure S5 Tables summarizing the scaling description of inflammation (upper) and modified ashcroft (down) scores.
